# Guidelines for Mountain Rescue During the COVID-19 Pandemic: Official Guidelines of the International Commission for Alpine Rescue

**DOI:** 10.1089/ham.2021.0032

**Published:** 2021-06-23

**Authors:** Steven Roy, Inigo Soteras, Alison Sheets, Richard Price, Kazue Oshiro, Simon Rauch, Don McPhalen, Maria Antonia Nerin, Giacomo Strapazzon, Myron Allen, Alistair Read, Peter Paal

**Affiliations:** ^1^ Department of Critical Care Medicine, University of Calgary, Calgary, Canada.; ^2^ International Society for Mountain Medicine, Bern, Switzerland.; ^3^ Medical Commission of the International Commission for Alpine Rescue (ICAR MEDCOM), Zurich, Switzerland.; ^4^ Emergency Medical System, University of Girona, Catalonia, Spain.; ^5^ Emergency Medicine, Boulder Community Health, Boulder, Colorado, USA.; ^6^ School of Medicine, University of Colorado, Aurora, Colorado, USA.; ^7^ LandSAR, Christchurch, New Zealand.; ^8^ Mountain Medicine, Research, and Survey Division, Department of Cardiovascular Medicine, Hokkaido Ohno Memorial Hospital, Sapporo, Japan.; ^9^ Department of Anaesthesiology and Intensive Care Medicine, F. Tappeiner Hospital, Merano, Italy.; ^10^ Institute of Mountain Emergency Medicine, Eurac Research, Bolzano, Italy.; ^11^ Department of Surgery, University of Calgary, Calgary, Canada.; ^12^ Jose Ramon Morandeira Mountain Medicine Association-CUEMUM, Chía, Spain.; ^13^ Corpo Nazionale Soccorso Alpino e Speleologico, National Medical School (CNSAS SNaMed), Milan, Italy.; ^14^ National Ski Patrol, Lakewood, Colorado, USA.; ^15^ Terrestrial Commission of the International Commission for Alpine Rescue (ICAR), Zurich, Switzerland.; ^16^ Mountain Rescue, England and Wales, Tamworth, United Kingdom.; ^17^ Department of Anaesthesiology and Intensive Care Medicine, Hospitallers Brothers Hospital, Paracelsus Medical University, Salzburg, Austria.; ^18^ Austrian Board for Mountain Safety, Innsbruck, Austria.; ^19^ Austrian Society of Mountain and High Altitude Medicine, Mieming, Austria.

**Keywords:** COVID-19, Emergency Medicine, infections, mountain rescue, SARS-CoV-2, technical rescue

## Abstract

Roy, Steven, Inigo Soteras, Alison Sheets, Richard Price, Kazue Oshiro, Simon Rauch, Don McPhalen, Maria Antonia Nerin, Giacomo Strapazzon, Myron Allen, Alistair Read, and Peter Paal. Guidelines for mountain rescue during the COVID-19 pandemic: official guidelines of the International Commission for Alpine Rescue. *High Alt Med Biol*. 22: 128–141, 2021.

***Background:*** In mountain rescue, uncertainty exists on the best practice to prevent coronavirus disease 2019 (COVID-19) transmission. The aim of this work was to provide a state-of-the-art overview of the challenges caused by the COVID-19 pandemic in mountain rescue.

***Methods:*** Original articles or reviews, published until December 27, 2020 in Cochrane COVID-19 Study Register, EMBASE, PubMed, and Google Scholar were included. Articles were limited to English, French, German, or Spanish with the article topic COVID-19 or other epidemics, addressing transmission, transport, rescue, or cardiopulmonary resuscitation.

***Results:*** The literature search yielded 6,190 articles. A total of 952 were duplicates and 5,238 were unique results. After exclusion of duplicates and studies that were not relevant to this work, 249 articles were considered for this work. Finally, 72 articles and other sources were included.

***Conclusions:*** Recommendations are provided for protection of the rescuer (including screening, personal protective equipment [PPE], and vaccination), protection of the patient (including general masking if low risk, specific PPE if high risk), equipment hygiene (including disinfection after every mission), use of single-use products, training and medical measures under COVID-19 precautions, and psychological wellbeing of rescuers during the COVID-19 pandemic. Adapted COVID-19 precautions for low-and-medium-income countries are also discussed.

## Introduction

Outdoor sports and mountain rescue have been severely affected by the coronavirus disease 2019 (COVID-19) pandemic. Early in 2020, first in East Asia and soon thereafter in Europe and America, countries introduced lockdowns to suppress the steep rise in infections. By mid-March 2020 in Europe and the United States, most ski resorts were closed. Dwindling mountain recreationalists resulted in fewer accidents. By June 2020, numbers of visitors in mountain areas bounced back and surged even above previous years. Consequently, mountain accidents have increased to record levels (BBC, 2020; BCSARA, 2020; Gawel, 2020; Hu, 2020; Scottish Mountain Rescue, 2020). In the northern hemisphere, the winter season has been severely affected by the pandemic, several skiing areas were shut down (e.g., Germany and Italy) or experienced a massive drop in visitors because of travel restrictions (e.g. Austria and Switzerland). It remains to be seen how the incidence of mountain accidents develops in the coming seasons.

In mountain rescue, uncertainty exists regarding best practice to prevent COVID-19 transmission. Regionally developed guidelines are mixed consensus and evidence-based owing to the limited data available for the mountain-specific context. The aim of this review was to provide a state-of-the-art overview on the challenges caused by the COVID-19 pandemic in mountain rescue. Guidelines are provided and graded according to evidence. In the following, suspected or confirmed COVID-19 patients will be named COVID-19 patients.

## Methods

A group of members of the air, medical, and terrestrial commissions of the International Commission for Alpine Rescue (ICAR) was summoned by a call of interest. Rescuers from ICAR member organizations were surveyed to determine areas where rescuer guidance would be particularly valuable. Subsequently, pertinent COVID-19 literature was identified and selected through a scoping literature review in accordance with Preferred Reporting Items for Systematic reviews and Meta-Analyses extension for Scoping Reviews (PRISMA-ScR) guidelines (Tricco et al., 2018). This method was chosen as it was felt to be most appropriate for identifying and mapping the available evidence on this quickly evolving topic.

Two authors (S.R., P.P.) searched the literature with Cochrane COVID-19 Study Register, EMBASE, PubMed, and Google Scholar, using the following terms alone and in combination: “COVID-19,” “emergency medical services,” “mountain rescue,” “out-of-hospital,” “SARS-CoV-2.” The composite search string used was “emergency medical services” OR “out-of-hospital” OR “pre-hospital” OR “rescue” OR “transport” for the Cochrane Register, ((“COVID-19”) OR (“SARS-CoV-2”) OR (“pandemic”)) AND ((“emergency medical services”) OR (“out-of-hospital”) OR (“pre-hospital”) OR (“mountain rescue”) OR (“transport”)) for EMBASE and PubMed and ((“COVID-19”) OR (“SARS-CoV-2”) OR (“pandemic”)) AND (“mountain rescue”) for Google Scholar. The literature review was updated to include all publications until December 27, 2020. The bibliographies of included articles were hand-searched for additional articles that had not been identified through the literature search. Finally, a personal search among authors and a web search were performed to identify regional or national mountain rescue guidelines for the COVID-19 pandemic.

Abstracts were imported into Covidence systematic review software (Veritas Health Innovation, Melbourne, Australia). Abstracts were independently screened by two authors to identify articles that met the following inclusion criteria: (1) original article or review; (2) published in English, French, German, or Spanish; (3) article topic was COVID-19 (any setting), pandemics (any setting), or other infectious outbreaks (mountain setting); and (4) addressed aspects of transmission, transport, training, rescue, or cardiopulmonary resuscitation (CPR). Abstracts that met the initial screening criteria underwent subsequent full-text review and those that did not meet the screening criteria were excluded. Full-text review was then conducted to determine which articles were relevant. These articles were provided to the guidelines panel for consideration. The panel used a consensus approach to develop recommendations and graded each recommendation according to criteria stipulated by the Grading of Recommendations Assessment, Development and Evaluation guidelines (Table 1) (Guyatt et al., 2006). In the discussion we present the evidence informing our recommendations, supplemented by expert opinion and experience. Afterward the recommendations to the pertinent section are given in separate tables.

**Table 1.** Classification Scheme for Grading Evidence

Grade 1A	Strong recommendation, high quality evidence, benefits clearly outweigh risks and burden or vice versa
Grade 1B	Strong recommendation, moderate-quality evidence, benefits clearly outweigh risks and burdens or vice versa
Grade 1C	Strong recommendation, low-quality or very low-quality evidence, benefits clearly outweigh risks and burdens or vice versa
Grade 2A	Weak recommendation, high-quality evidence, benefits closely balanced with risks and burdens
Grade 2B	Weak recommendation, moderate-quality evidence, benefits closely balanced with risks and burdens
Grade 2C	Weak recommendation, low-quality or very low-quality evidence, uncertainty in the estimates of benefits, risks and burden; benefits, risk and burden may be closely balanced

## Results

The literature search yielded 6,190 articles. A total 952 were duplicates and 5,238 were unique results. In the initial abstract screening, 273 studies met the inclusion criteria for full-text review and 4,965 articles did not meet the inclusion criteria and were excluded. Full-text review revealed 6 duplicates that were not identified automatically by our systematic review software, leaving 267 original articles to review. On full-text review, 18 articles did not meet the inclusion criteria and were excluded. The remaining 249 articles were included for consideration. A total of 72 articles and other sources were referenced in the final manuscript. Selected results of the rescuer survey are included in the Supplementary Appendix S1.

## Discussion and Recommendations

### Specific challenges in mountain rescue related to the COVID-19 pandemic

Since the early stages of the pandemic, during the warm months rescue call volumes have increased substantially, straining resources for small rescue teams and creating constraints around training and staffing. In mountain compared with urban rescue, staff and equipment are limited, more so in terrestrial than in airborne missions. During terrestrial rescue, substantial physical efforts may be necessary. Often, mountain rescue has to be provided in an austere environment with limited material and staff in close proximity between victims and rescuers. The risk of contracting COVID-19 outdoors is thought to be low when acting prudently (Qian et al., 2021), but care should still be taken when in close contact with patients and other rescuers. During technical rescue activities, some forms of personal protective equipment (PPE) may introduce unacceptable communications or visibility risks or limit exertion and performance. Wet weather and sweating may decrease protection of PPE and threaten the safety of mountain rescuers from fogging of eye protection. Specific recommendations are given in Table 2.

**Table 2.** Challenges in Mountain Rescue Related to the Coronavirus Disease 2019 Pandemic

No.	Recommendation	Grade
1	The risks of exposure to COVID-19 during mountain rescue should be identified.	1C
2	COVID-19 precautions may complicate, distract from or increase the general risks of mountain rescue. Maintain situational awareness.	1C

COVID-19, coronavirus disease 2019.

### Rescuer safety and infection prevention

The severe acute respiratory syndrome coronavirus type 2 (SARS-CoV-2) is mainly transmitted through respiratory droplet. The evolving science on COVID-19 transmission is discussed in detail elsewhere (Berlin et al., 2020; Gandhi et al., 2020; Wiersinga et al., 2020). Specific recommendations for rescuer safety and infection prevention are given in Table 3.

**Table 3.** Rescuer Safety and Infection Prevention

No.	Recommendation	Grade
General principles		
3	Any rescuer that is ill with symptoms of COVID-19 or other illness should not respond to missions and should not participate in training exercises.	1C
4	If a rescuer has had a high-risk exposure or COVID-19-positive test, they should quarantine according to regional or national guidelines before returning to duties.	1C
5	The risks of exposure to COVID-19 during mountain rescue should be identified and managed in the context of the general risks of mountain rescue.	1C
6	Mountain rescuers should be trained in the selection, application, and removal of appropriate PPE.	1C
Specific principles: masks and face coverings		
7	Rescuers should wear a medical grade surgical mask covering mouth and nose whenever in close contact with other rescuers or patients (2 meters), provided that it does not introduce other unacceptable safety risks.	1A
8	Rescuers who cannot maintain a 2-meter distance from patients or who must perform aerosolizing procedures should wear masks with N95/FFP2 or higher ratings.	1A
9	Masks with expiratory valves are not recommended as they do not prevent aerosolized particles from spreading.	1B
10	When working in high-winds and rotor wash, masks should be selected to ensure that the mask cannot be bypassed when air movement comes from behind the wearer.	2C
11	Water resistant masks should be selected for rescues where precipitation or spray are likely to be encountered.	2C
12	Wet masks should be replaced as soon as feasible.	2C
Eye and hand protection		
13	Eye protection should be worn by providers who are involved in patient care or in close proximity	1C
14	Medical grade, waterproof gloves should always be used when providing patient care.	2C
15	Hand washing should be performed before donning and after doffing PPE, before and after patient care when gloves are unavailable, before and after eating or touching one's face, after blowing your nose, coughing, or sneezing, and after cleaning or disinfecting equipment. If soap and water are not readily available, use a hand sanitizer that contains at least 70% alcohol.	1C
Equipment cleaning and return to service		
16	Rescuers should disinfect potentially contaminated rescue equipment with soap and water, 70% isopropyl alcohol, or viricidal agent that does not damage the material.	1B
17	Clothing should be doffed and washed with detergent or technical wash as appropriate.	1C
18	Ropes, cord, webbing and other load-bearing soft goods should be cleaned according to manufacturers' recommendations.	1C
19	For equipment that can't be cleaned for logistical or safety reasons, we recommend quarantine of the equipment for at least 72 hours.	2C
Vaccination and prophylaxis		
20	Vaccinations are recommended for eligible rescuers.	1A
21	Vaccination may not remove the need for individual and team safety precautions recommended above.	2C
Psychological support		
22	Mountain rescue teams should encourage and promote communication and training activities that address rescuer stress and promote resilience.	2C

PPE, personal protective equipment.

#### General principles

Search and Rescue (SAR) is an essential function provided to recreationalists, workers, and travelers throughout the world in mountain and wilderness areas. SAR teams need to maintain training and fitness to be ready for these demanding activities and local COVID-19 infection rates affect their ability to train and respond. Following regional and national COVID-19 prevention guidelines is important to reduce community transmission. Rescuers who are at high risk for COVID-19 complications need to consider their suitability for the job during this pandemic. Although there are ways to reduce risk of viral exposure when caring for patients, it cannot be eliminated entirely.

Preventing viral transmission during SAR activities during the pandemic is vital and challenging. Best practices for PPE evolve, but as essential workers, it is recommended that rescuers protect themselves, maintain social distance whenever possible, provide facemasks for patients and bystanders, and limit the number of rescuers exposed to any possible SARS-CoV-2 source. Separating rescue and incident management teams into working bubbles provides a degree of infection prevention (Shaw et al., 2020). This may prevent entire teams from being taken out of service for quarantine after potential exposures. Detailed logs of personnel attendance at training and missions, including outside agencies, must be maintained for possible future contact tracing.

Prevention includes maintaining social distance at trailheads and staging areas, limiting the number of rescuers in huts, tents, snow shelters, caves, crevasses, aircraft, vehicles, and gear caches (Knibbs et al., 2012). Scenes should be approached with at least 2 m of separation between individuals on trails whenever possible. The minimum airborne-precaution PPE includes gloves, long-sleeves, and pants, a N95 or FFP2 respirator or equivalent, and eye and face protection. Wearing a facemask has been shown to reduce the dispersion of respiratory secretions and, therefore, reduces risk of SARS-CoV-2 transmission. Wearing PPE of appropriate type and fit can be cumbersome when doing strenuous activity leading to fogging of eye protection, difficulty breathing, overheating, decreased exercise capacity, and problems in verbal communication.

Rescuers may lower masks on trails when social distancing can be maintained. Similarly long sleeves, pants, and eye protection can be kept off until it becomes necessary to get close to patients, teammates, or other subjects on scene. Once rescuers are within 2 m of the patient, PPE should be maintained on rescuers unless it compromises the safety of the operation. Patients must have facemasks placed as soon as possible, a higher protection level should be considered (e.g., N95/FFP2), if COVID-19 is suspected or confirmed. Rescuers should stay upwind of the patient.

If a rescuer has had a high-risk exposure or COVID-19 positive test, they should quarantine according to regional or national guidelines before returning to duties (CDC, 2020a, CDC, 2020b; WHO, 2020a). Care should be taken to avoid cross-contamination and cleaning protocols for rescuer and team equipment should include equipment cleaning at end of mission. Rescuers should carry spare PPE and a biohazard disposal bag. During rescue and when donning and doffing PPE the buddy check system should be endorsed. The waste produced during the rescue has to be managed and disposed of properly.

#### Specific principles

##### Masks

Masks are highly effective in reducing SARS-CoV-2 transmission and are advised (Chu et al., 2020; Leung et al., 2020; Liang et al., 2020). In particular, surgical and medical grade masks demonstrate superior performance in the clinical environment and are preferred over cloth masks, bandanas, or commercially available neck gaiters. Nonsurgical masks should have at least two layers of fabric (CDC, 2020c), although some authorities suggest three layers (WHO, 2020b) if medical grade masks are unavailable. A proper fit over the nose, mouth, and chin are necessary for any type of mask. When aerosol-generating procedures (AGP) such as suction, high flow oxygen, intubation, or chest compressions are needed, rescuers attending to a patient should use at least a N95/FFP2 respirator to prevent viral spread. Simple surgical masks are reported to be both inferior in risk reduction (MacIntyre et al., 2013; Chou et al., 2020; Chu et al., 2020; Wang et al., 2020) and equivalent (Radonovich et al., 2019; Bartoszko et al., 2020; Long et al., 2020) when compared with N95/FFP2 respirators. Medical masks and respirators are critical supplies that should continue to be reserved for health care workers and other medical first responders depending on local supply. Masks with expiratory valves are not recommended, as they do not prevent aerosolized particles from spreading. Small children, patients with trouble breathing, unconscious, or incapacitated patients may require careful consideration around mask utilization to reduce potential viral spread.

Masks and respirators are associated with increased respiratory rate and perceived effort (Jones, 1991), impaired cardiopulmonary exercise capacity (Chen et al., 2016; Fikenzer et al., 2020; Epstein et al., 2021), potential user discomfort (Lan et al., 2020; Mushtaq et al., 2021), and may interfere with communication (Burton et al., 2020). Some studies suggest that physically demanding tasks may be impeded by the mask (Chen et al., 2016; Fikenzer et al., 2020). Others suggest that while perceived respiratory effort may be increased, vigorous exercise and CPR quality are not affected by masking (Hopkins et al., 2020; Kienbacher et al., 2021). Mask performance may be reduced by moisture from sweating or heavy breathing. Medical masks are designed for a relatively dry, indoor, controlled environment and replacements could be necessary for some environments. Medical masks lose their physical integrity or their ability to effectively prevent transmission of microbes when wet (Andersen, 2019).

##### Eye protection

Eye protection when performing SAR activities is generally appropriate to prevent injury, snow blindness, and so on. Eye protection, especially “wrap around” or goggle type eyewear may confer additional benefit to the mask (Lindsley et al., 2020) in reducing the risk of viral transmission (Chu et al., 2020; WHO, 2020c). Face shields are an option and may be part of helmet assembly facilitating ease of use.

##### Gloves

Medical grade, waterproof gloves should be used when providing patient care. Availability of gloves is generally good, and cost is low. Some gloves used by rescuers for rope work and warmth do not provide the same level of protection but may be placed over medical gloves or may replace medical gloves once patient care is complete.

##### Clothing/body covering

Because medical gowns are not practical in alpine rescue, clothing that covers the full upper and lower torso and limbs may be used to avoid contact transmission. Watertight long-sleeved garment and trousers are suitable. One-piece suits (e.g., Tyvek^®^ 800J) may also be worn; however, rescuers should be mindful of their condition during hot or strenuous activities as overheating may occur. Gowns can be used during airway management to protect providers' skin and clothing from contamination. Be aware of variable permeability of gowns depending on rating. Clothing should be doffed and stored in a reusable or disposable bag at the end of a mission. A change of clothes and doffing bags for contaminated outerwear should be available at the end of a mission to prevent vehicle contamination.

##### Equipment cleaning and return to service

Once used, equipment with risk of contamination should be decontaminated before return to service. Treatment with soap and warm water or isopropyl alcohol are effective in removing detectable virus from equipment (Chin et al., 2020; Pradhan et al., 2020) and may be used when they will not damage equipment. Ropes, cord, webbing, and other life-safety soft goods should be cleaned according to manufacturers' recommendations. Smooth hard surfaces appear to harbor detectable viral material longer than textiles, but are also more easily cleaned (Chin et al., 2020; van Doremalen et al., 2020). For equipment that cannot be cleaned for logistical or safety reasons, quarantine of the equipment has been proposed (Black Diamond Equipment, 2020; DMM Wales, 2020; Greatbatch et al., 2020; Kong Italy, 2020; Maxim Ropes, 2020; Petzl America, 2020; Singing Rock, 2020). Recommendations for quarantine of equipment vary from 3 to 7 days (BEAL, 2020; Black Diamond Equipment, 2020; DMM Wales, 2020; Kong Italy, 2020; Mammut, 2020; Petzl America, 2020; Singing Rock, 2020). Quarantine of equipment results in equipment being inaccessible for rescue, and therefore may be impractical (Greatbatch et al., 2020).

##### Vaccination

Vaccinations are now available against the most common strain of COVID-19 (Sahin et al., 2020; Baden et al., 2021), although individual strain prevalence is changing. Vaccinations should be offered according to vaccine manufacturers guidelines and national guidelines as early as possible to all rescuers involved in patient care. Local and regional protocols may determine availability.

##### Psychological support

The global pandemic has caused considerable stress to many rescuers and nonrescuers alike. Acute stress injury may exacerbate preexisting anxiety, depression, and other psychological illnesses and result in more serious stress disorders (Miguel-Puga et al., 2021). Education and increased awareness of stress injuries could be valuable to build resilience and prevent adverse psychological consequences in mountain rescuers (Krystal, 2020).

### Terrestrial and airborne rescue

Specific recommendations are given in Table 4.

**Table 4.** Treatment and Rescue Recommendations

No.	Recommendation	Grade
22	When approaching a patient during the pandemic, droplet-precaution PPE should be used routinely. When performing potentially aerosol-producing procedures, airborne-precaution PPE should be used.	1C
23	A surgical mask should be placed on the patient immediately upon contact and to keep that in place throughout medical maneuvers and transport.	2C
24	Approaching a casualty during a technical operation, rescuers should ideally don PPE appropriate for close interactions with the patient (e.g., facemask and fluid-resistant N95/FFP2 respirator), provide a conscious patient with a facemask and gloves, and maintain an at least 2 meter distance where possible until removal to a safe place for medical care.	2C
25	If the patient needs supplemental oxygen, an oxygen mask can be worn over the top of a surgical mask without compromising the FiO_2_.	2C
26	Airborne-precaution PPE should be used while performing bag-valve mask ventilation.	2C
27	A high-efficiency particulate air (HEPA) filter or a heat and moisture exchanger (HME) filter should be placed between the self-inflating bag and the mask.	2C
28	A two hand technique should be used to hold the mask to ensure a good seal of the mask.	2C
29	For tracheal intubation, videolaryngoscopy is preferred over direct laryngoscopy, because the airway operator will be more distant to the patient's mouth. The most experienced operator should intubate as prolonged airway management time likely correlates with a higher viral exposure.	2C
30	Paralytics should be part of the rapid sequence induction of anesthesia to avoid coughing and gagging and to optimize tracheal intubation conditions.	2C
31	Following intubation, routine airway suctioning should be avoided unless absolutely needed as suction is an AGP, and it can also stimulate coughing if the patient is not adequately sedated or paralyzed.	2C
32	Applying defibrillator pads and delivering a shock from an AED/defibrillator is unlikely to be an aerosol-generating procedure and can be undertaken with the rescuer wearing droplet-precaution PPE only.	2C
33	Chest compressions have the potential to generate aerosols and airway interventions are AGP. Rescuers should therefore wear airborne-precaution PPE before starting chest compressions or perform airway management.	1C
34	Cardiac arrest is recognized by looking for the absence of signs of life and the absence of normal breathing. To minimize the risk of infection, the airway should not be opened, and the rescuers' face should not be placed next to the victims' mouth and/nose.	2C
35	Chest compressions and ventilation with a bag-mask and oxygen should be performed at a 30:2 ratio, pausing chest compressions during ventilations to minimize the risk of aerosol.	2C
36	Rescuers less skilled or uncomfortable with bag-mask ventilation should not provide bag-mask ventilation because of the risk of aerosol generation. They should place an oxygen mask on the patient's face, give oxygen and provide compression-only CPR.	2C
37	For teams performing bag-mask ventilation, one rescuer will hold the mask with a two hand-technique, the second rescuer switches between 30 chest compressions and 2 bag ventilations.	2C
38	If a defibrillator is immediately available, a shock should be delivered if the rhythm is ventricular fibrillation or pulseless ventricular tachycardia (VF/pVT). If the patient remains in VF/pVT after the first shock, and if the rescuer is wearing airborne-precaution PPE, chest compressions should be initiated. If the rescuer is not wearing airborne-precaution PPE, up to two additional shocks should be given if VF or pVT persists, and while other rescuers are putting on airborne-precaution PPE.	2C
39	Rescuers experienced in airway management should insert a supraglottic airway or intubate the trachea early so that the period of bag-mask ventilation is minimized.	2C
40	If a supraglottic airway has been inserted, a 30:2 chest compression ventilation ratio should be used, pausing the chest compressions to enable ventilation. This will minimize the risk of aerosol generation caused by air leaking from the seal between the supraglottic airway and the larynx.	2C
41	Stopping CPR early should be considered if not treatable reversible causes of cardiac arrest have been addressed.	2C
42	If there is a need for prolonged CPR, a mechanical chest compression device should be used by rescuers who are familiar with its use.	2C
43	During packaging of a patient without confirmed COVID-19, droplet-precaution PPE should be used routinely.	2C
44	During splinting and immobilization of a patient without confirmed COVID-19, droplet-precaution PPE should be used routinely.	2C
45	Routes of fluid and drug administration that increase risk of rescuer exposure to aerosols or mucous membranes (e.g., direct oral or transmucosal administration) should be avoided where feasible, based on patient status and logistical challenges.	2C

AED, automatic emergency defibrillator; AGP, aerosol-generating procedure; CPR, cardiopulmonary resuscitation; VF/pVT, ventricular fibrillation or pulseless ventricular tachycardia.

#### Patient treatment

During the COVID-19 pandemic it must be assumed that any patient is potentially infected with SARS-CoV-2. Asymptomatic individuals can transmit the virus. Therefore, precautionary measures have to be adopted to reduce risk of infection. Some have suggested that during ground rescue operation, screening of any conscious patient with a COVID-19 questionnaire should be performed (Massullo et al., 2021). Known or likely COVID-19-positive patients should be treated with extra caution. The consensus of our group is that every patient is considered as potentially COVID-19 positive. Because the SARS-CoV-2 infection rates vary worldwide, the recommendation regarding PPE while treating patients without confirmed or likely COVID-19 should be adjusted according to local protocols and regional risk assessment (Chu et al., 2020).

**FIG. 1.**
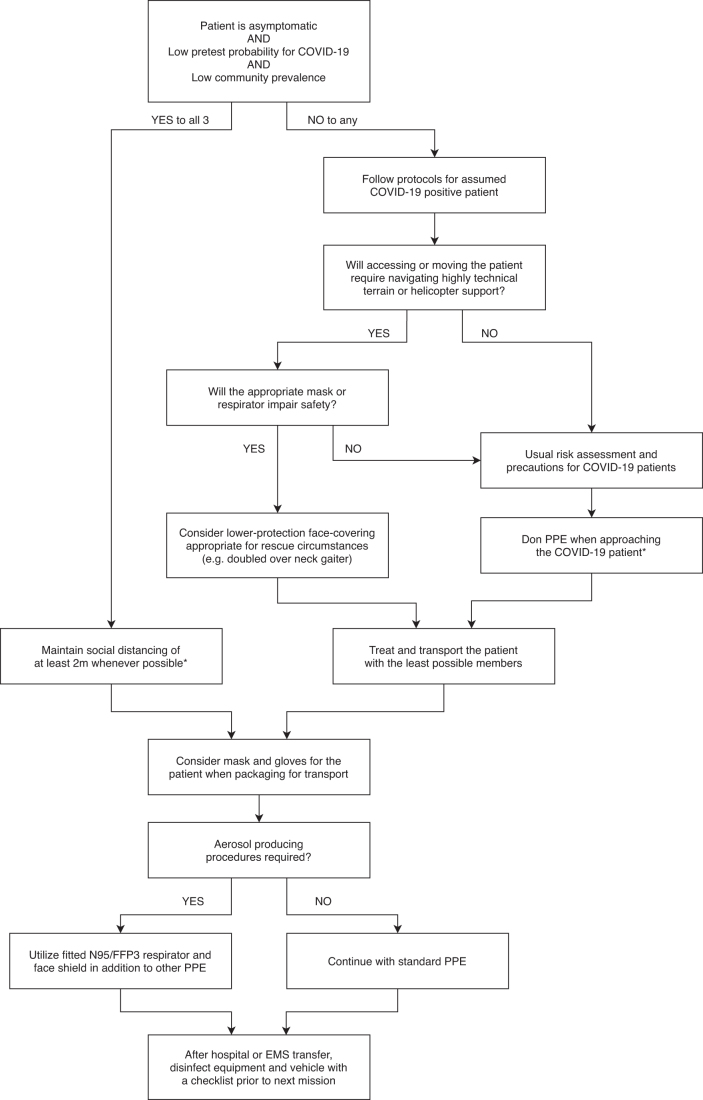
Treatment algorithm in mountain rescue for an ill or injured patient during the COVID-19 pandemic. *In accordance with any local and national guidelines. COVID-19, coronavirus disease 2019.

During difficult technical operations (e.g., rope maneuvers, hoisting, canyoning rescue), it may be challenging to keep PPE in place. Rescue organizations should choose PPE compatible with the use of technical equipment such as helmets and harnesses. Their use might be limited during complex technical rescues and in harsh environments where mountain rescuers are exposed to factors like water, wind, rain, and snow. Rescuer exercise capacity and comfort may be impaired by disposable medical respirators during exercise and can increase CO_2_ rebreathing (Chen et al., 2016; Fikenzer et al., 2020; Epstein et al., 2021; Mushtaq et al., 2021). Optimal face coverings depend on the patient, terrain, and specific rescue characteristics. PPE should be selected to balance performance limitation and impaired communication with the benefit of reducing potential viral exposure. An approach to selecting appropriate face coverings is given in Figure 1.

#### Airway management

Airway management is an AGP, it should be avoided whenever possible. Full PPE should be used if airway management is required. Specific guidelines have been published on airway management. In a hypoxic patient with sufficient respiratory effort, a nasal cannula may be used with an N95/FFP2 mask on top. Only a negligible difference in FiO_2_ was found when an oxygen mask was placed over a surgical mask (Binks et al., 2020).

#### Basic life support and advanced life support

CPR is an AGP. CPR has to include COVID-19 precautions. The International Liaison on Committee on Resuscitation (ILCOR) and the European Resuscitation Council (ERC) published COVID-19 guidelines for basic life support and advanced life support (Nolan et al., 2020; Perkins et al., 2020).

#### Other medical maneuvers

#### Insulation

A casualty should be protected against further heat loss with a whole-body insulation covered by a vapor barrier outer shell (Paal et al., 2016). Placement of insulation requires being in close proximity with the casualty, but it is not considered an AGP.

#### Management of fractures and dislocations

Splinting and immobilization are common on-site medical interventions in mountain rescue operations (Ellerton et al., 2009). Splinting requires close contact with the casualty, but this is not an AGP.

#### Fluids and drugs

Intravenous access remains the cornerstone of drug administration in emergencies. Establishing intravenous access may be particularly difficult or impossible during mountain rescue operations in casualties with a collapsed vascular bed (e.g., in cardiac arrest or hypovolemic shock). A recent review compared the use of intravenous and intraosseous access in providers wearing high-precaution PPE, and suggested that in this context intraosseous access is achieved faster (Drozd et al., 2021). Intraoesseous access may have limitations during prolonged rescues or in aqueous environments (Strapazzon et al., 2018). Additional routes of drug administration have been suggested in mountain rescue. Intranasal, transmucosal, and oral are minimally invasive and are generally good choices for rescue in harsh environments. However, in casualties with suspected or confirmed COVID-19, intramuscular and subcutaneous administration may be preferable routes of administration compared with oral and transmucosal routes as they avoid potential exposure to mucous membranes.

#### Handling bodies of deceased persons with COVID-19

It is thought that transmission of SARS-CoV-2 is possible through contact with contaminated surfaces or direct contact with human remains or bodily fluids where the virus is present (Yaacoub et al., 2020). At present, there is no evidence of transmission of SARS-CoV-2 through the routine handling of bodies of deceased persons, and the potential risk of COVID-19 transmission in this setting is considered low (Yaacoub et al., 2020). Bodies should be packaged for transport in a manner that minimizes the risk of spray of bodily fluids. Postmortem examinations should be undertaken with PPE appropriate to the type of examination being undertaken (Table 5).

**Table 5.** Handling of a Deceased Person

46	During standard handling, the risk associated with transmission of droplets or aerosol from the airways of the deceased is considered low and gloves, a long-sleeved apron, and a fluid-resistant surgical mask should be used.	2C
47	During postmortem examinations, aerosol-generating procedures or procedures that can lead to splashes carry a potential risk and require airborne-precaution PPE.	1C
48	Both the movement and handling of the body should be kept to a minimum.	2C
49	All lines, catheters, and other tubes should not be removed from the deceased body as this might increase spilling of body fluids (e.g., blood) and interfere with a forensic examination. Puncture holes should be disinfected.	2C
50	Body fluids leaking from orifices should be contained.	2C
51	The body should be wrapped in cloth; body bags may be used for other reasons (e.g., excessive body fluid leakage).	2C

### Transportation

Specific recommendations are given in Table 6. The integration of mountain rescue, emergency medical services (EMS), and hospital services is crucial to ensure appropriate management and safe transport of suspected or confirmed COVID-19 patients (Bredmose et al., 2020). Responsibilities may differ, good communication is key; COVID-19 patients have to be identified in a timely manner. Mountain rescue teams should be prepared to safely transport COVID-19 patients. The transport of COVID-19 patients should be conducted by experienced teams utilizing appropriate equipment (Bredmose et al., 2020).

**Table 6.** Transportation Challenges

No.	Recommendation	Grade
52	Integrate mountain rescue, emergency medical services (EMS), and hospital services to ensure appropriate management and safe transport of suspected or confirmed COVID-19 patients.	2C
53	The transport of COVID-19 patients should be conducted by experienced teams utilizing appropriate equipment.	2C
54	Consider the use of “isolation pods” with appropriate air filtration where cabin separation is not possible.	2C
55	Minimize members involved in patient transport and care. Where feasible, employ transport techniques and equipment, such as wheeled litters, that minimize the total workforce required.	2C
56	Patients should wear PPE including surgical mask and nonsterile gloves. Consider packaging the patient to reduce infectivity, i.e., wrapping the patient into a plastic sheet, before transport.	1C
57	Immediately after a patient transport, all exposed surfaces of a vehicle should be disinfected from top to bottom in a standardized systematic process before the vehicle returns to normal operational duties.	2C
58	A checklist of areas to be cleaned can be used to improve coverage.	2C
59	Disinfection should be done by the staff involved in the patient transport. Staff cleaning the vehicle should wear PPE for infection precautions.	2C
60	Cleaning is specific to each vehicle type. If the vehicle is cleaned at the garage or hangar, a PPE disposal system must be present.	2C
61	Inside a vehicle, the minimum number of persons should be present to avoid incidental contact.	2C

Some organizations may consider the use of “isolation pods” with appropriate air filtration where cabin separation is not possible. Minimize members involved in patient transport and care (Albertch et al., 2020).

#### Patient packaging consideration

Patients should wear PPE including surgical mask and nonsterile gloves. Consider packaging the patient for technical rescue even if the injury does not require it to reduce the risk of transmission. Packaging means wrapping the patient into a plastic sheet before placing him on the stretcher (Bredmose et al., 2020).

#### Vehicle decontamination after a suspected or confirmed COVID-19 case

Immediately after a patient transport, all exposed surfaces of the vehicle should be disinfected before it returns to normal operational duties. Universal sanitizing wipes or a chlorine-based solution at 1,000 parts per million (or approved equivalent) should be used. When AGP have been performed, work from top to bottom in a standardized systematic process. A checklist of areas to be cleaned can be used to improve coverage (Bredmose et al., 2020). Any exposed equipment, which was not sealed off during transport, requires disinfection. Finally, the vehicle floor requires cleaning and disinfection (CDC, 2020d).

Disinfection should be performed by the staff involved in the patient transport. Staff cleaning the vehicle should wear PPE for infection precautions (including a long-sleeved impervious gown, surgical mask, goggles, and nonsterile gloves). Therefore, it may be prudent to clean the vehicle at the receiving hospital, before doffing their PPE (especially with PPE shortages). Cleaning is specific to each vehicle type and includes wiping of all surfaces with a cleaning product followed by a disinfectant or a combined product. If the vehicle is cleaned at the garage or hangar, a PPE disposal system must be present (CDC, 2020e).

#### Airframes

In most medical helicopters used in mountain rescue, the cabin is a single, shared environment between pilot, medical staff, and patient. In such airframes, it is not possible to isolate the pilot from the patient compartment. The helicopter staff may not doff PPE until the cabin has been decontaminated. It is essential to consult aircraft engineers, as some cleaning products may damage the aircraft. The aircraft will require additional down time to be adequately cleaned and dried before a subsequent mission. Wiping the cabin is estimated to take an hour; additional time may be needed for fumigation and drying.

#### Ground vehicles

Isolation of the patient compartment from the passenger compartment and filtration of the air may be possible in ground transport vehicles (Osborn et al., 2020). Separation creates a safer environment for the rescue teams. Limit the number of providers in the patient compartment to essential personnel to minimize possible rescuers exposures. Ground vehicles will be decontaminated after transfer of care at the receiving facility. A minimum number of persons should decontaminate the inside of a vehicle to avoid incidental contamination (WHO, 2020d).

### Mountain rescue training

Specific recommendations are given in Table 7.

**Table 7.** Mountain Rescue Training Challenges

No.	Recommendation	Grade
62	Mountain rescue teams should regularly practice specific COVID-19 PPE procedures and alternative rescue techniques during training operations.	2C
63	COVID-19 testing for team members should be considered before training operations on the advice of the team medical director.	2C
64	Rescue teams should review and prioritize essential training operations during the pandemic to optimize training while reducing risk of COVID-19 exposure. Consider smaller training group numbers.	2C
65	Rescue teams need additional funding, PPE supplies, personnel and support to conduct training operations during the pandemic.	2C
66	Team members should be excused from training and supported if they are unwell, have had a recent COVID-19 exposure or if they or their family members have medical concerns that predispose to serious COVID-19 complications.	2C

#### General challenges

Recommendations around rescue team training are based largely on rescue team members' comments and observations. The risk of COVID-19 exposure during mountain rescue training has generally made all training exercises slower, more difficult, and more expensive. Teams need to review and prioritize training activities over time to focus on essential training activities only for the time being. Alternative training methods can be used such as virtual first aid training and some training activities can be carried out with minimal group sizes or within training group bubbles. Records of training attendance should be retained to facilitate contact tracing in case a team member becomes symptomatic.

COVID-19 precautions including PPE use during training may introduce additional risks as discussed in previous sections. Team leaders should manage these risks in the context of other important training hazards.

Distancing during active mountain rescue training exercises can be challenging. Careful attention to precautions during travel and training should be practiced and evaluated as a normal part of training exercises. Training equipment should be handled, decontaminated, and/or quarantined according to team protocols. Teams may wish to modify rescue techniques in some situations (e.g., coaching a subject and their partners to self-package) and any modified procedures should be part of training exercises. Teams should receive regular medical direction regarding the use of COVID-19 testing within the group and vaccination to help guide training activities.

#### Specific training challenges

In the context of mountain rescue, AGP may be modified with appropriate medical direction. Modified procedures should be practiced during training using safe alternative training devices such as airway mannequins. First aid training can be enhanced to regularly practice PPE donning and doffing procedures with rescue clothing and gear, PPE use in the mountain environment, and to provide updated COVID-19-related medical information. Water and canyoning rescue may have to be substantially modified during the pandemic owing to the inherent problems of using PPE and training in wet environments where close contact and coughing are common and may be unexpected.

### Challenges in low-and-middle-income countries

Specific recommendations are given in Table 8. There is growing concern about the impact of the pandemic on low- and middle-income countries (LMICs). Of the 107 LMICs assigned a COVID-19 preparedness capacity level by the WHO, almost all (94) are at Level 3 (capacity is ≤60%) or below, with more than half (57) designated as Level 3, a third (36) as Level 2 (capacity ≤40%), and one as Level 1 (capacity ≤20%) (Kates et al., 2020). The WHO COVID-19 Strategic Preparedness and Response Plan acknowledges that rural and remote populations are vulnerable populations that deserve preparedness planning (WHO, 2021). Predicting the development of the outbreak and fighting the virus in the countryside is an ongoing challenge. In LMICs, most rescue operations are carried out by first responders or villagers from impoverished areas, before professional rescue arrives. The quality of provided medical support depends upon the available resources, including knowledge of COVID-19 prevention measures, PPE, EMS, testing kits, disinfectants and wastage treating, clean water. It has been stated that, “medical care in the wilderness is the art of the possible” (Zafren et al., 2012). This is true even more so when care has to be provided in an LMIC.

**Table 8.** Challenges in Low- and Middle-Income Countries

No.	Recommendation	Grade
67	Masks with at least two layers of washable and breathable fabric should be used. When resources are limited, use three layers of cloth for protection. Wash masks frequently and dry in the sun.	2C
68	Consider plexiglass shields to minimize exposure as well as plastic (or fabric) aprons.	2C
69	In the setting of significant PPE shortages, single-use PPE may be reused (ideally by the same person) after appropriate disinfection and quarantine as dictated by local, regional, and WHO guidelines.	2C

#### PPE for low resource countries

Rescue teams must adapt PPE according to the situation and implement means of circumstances (or fortune) to cover COVID-19 prevention measures. When rapid diagnostic testing is not possible, health care providers should consider recommending that the individual isolate for possible COVID-19 based on the current guidance and level of suspicion. Health care providers and rescuers should consider having patients wear a mask or cloth face covering and glasses for source control to prevent the spread of COVID-19. Being ill with an infectious disease, for example, malaria, dengue, or tuberculosis does not exclude coinfection with COVID-19. To cope with increased staffing demand during this pandemic, active recruitment and training of health care personnel should be carried out concurrently. For specific recommendations, see Table 7.

Masks with at least two layers of washable and breathable fabric reduce the spread of SARS-CoV-2 (CDC, 2020c). When resources are limited, WHO guidelines recommend three layers of cloth for protection (WHO, 2020b). Novel strategies, such as plexiglass shields, may be considered to minimize health care worker exposure and preserve PPE during transport as well as plastic or fabric aprons. Many of the disinfectants will not be available in the least developed countries. Bleach or alcohol after washing with soap and water are an alternative; however, most rescue equipment and mountain clothes may not be disinfected with bleach. Equipment that cannot be cleaned for logistical or safety reasons, may be quarantined when feasible (see Equipment Cleaning and Return to Service section).

During prolonged rescue and in case of extended or overnight rest, all individuals must respect as much as possible the hygiene, distancing, and barrier measures to avoid COVID-19 owing to the increase of time exposure, close situation, and closed place.

#### Nutrition during prolonged rescue

In 2001 the WHO introduced the Five Keys to Safer Food (World Health Organization, Department of Food Safety, Zoonoses and Foodborne Diseases, 2006). It is highly unlikely that people can contract COVID-19 from food or food packaging (WHO, 2020e). Good hygienic practices include the following: (1) mask, (2) hand hygiene, washing with soap and water for at least 20 seconds or frequent use of alcohol-based hand sanitizers; (3) respiratory hygiene (cover mouth and nose when coughing or sneezing; dispose of tissues and wash hands); (4) restricting nonessential physical contact; (5) cleaning and disinfection procedures for equipment, contact surfaces at least with soap; and (6) that patients should use their own utensils to drink and eat. In case of low disponibility, utensils are disinfected before being used by another person.

In LMICs, medical and rescue teams work with little equipment and often have to improvise. All solutions have to be assessed for feasibility, cost, and appropriateness. All planning, implemented steps, procedures should be documented safely for future endeavors. Every plan can fail thus a plan B should be ready as backup. LMICs need immediate support from the international community as they often lack the productive capacity and financial resources to obtain necessary health equipment.

### Knowledge gaps

The contagiosity of asymptomatic carriers has been documented but has not been quantified. The specific risk for transmission in air and on surfaces continues to be delineated. Infection prophylaxis after exposure, immunity after a previous COVID-19 infection and vaccination, vaccination modalities, treatment, and the hazard posed by SARS-CoV-2 mutations require further research.

### Outlook

Since the 1918/19 influenza pandemic, COVID-19 is the disease, which has claimed most victims in a given period. Unprecedently quick improvements in medical care have been achieved since COVID-19 appeared in late 2019. A return to a pre-COVID-19 life seems only possible with vaccinations. Experts have warned since several years of pandemics. It was not the question if/but when a pandemic would strike humankind. More than 20 pathogens are known to have a pandemic potential. Dense population, environmental change, globalization and habitat loss make the spread of further pathogens with pandemic potential more likely. The COVID-19 pandemic can teach the world several lessons, which should be learned and implemented before the next pandemic strikes humankind.

### Limitations

Several parts in this work are based on expert opinion and experience rather than pure science. Despite a low level of evidence, recommendations were graded as strong if they were considered important for safety reasons. This approach is consistent with other guidelines (Guyatt et al., 2006). Required social distancing and requirements for masks vary between areas. Knowledge of SARS-CoV-2 and COVID-19 is developing rapidly. The content of this article has to be seen within this rapidly changing science. Updates of this content are required as soon as major scientific breakthroughs are made.

## Conclusions

This article covers several important topics pertaining to mountain rescue and provides recommendation for mitigating key risks. Evidence-graded recommendations are provided for rescuer safety and infection prevention, protection of the patient, equipment disinfection and selection, training, and medical measures under COVID-19 precautions, and psychological wellbeing of rescuers during the COVID-19 pandemic. Adapted COVID-19 precautions for specific challenges in low-and-medium-income countries are also discussed. Finally, knowledge gaps are identified and an outlook is given.

## Supplementary Material

Supplemental data

## References

[B1] Andersen BM. (2019). Protection of upper respiratory tract, mouth and eyes. In: Prevention and Control of Infections in Hospitals: Practice and Theory. BM Andersen, ed. Springer International Publishing, Cham. pp. 129–146

[B2] Albrecht R, Knapp J, Theiler L, Eder M, and Pietsch U. (2020). Transport of COVID-19 and other highly contagious patients by helicopter and fixed-wing air ambulance: a narrative review and experience of the Swiss air rescue Rega. Scand J Trauma Resusc 28:40 10.1186/s13049-020-00734-9 PMC7222521 32410706

[B3] Baden LR, El Sahly HM, Essink B, Kotloff K, Frey S, Novak R, Diemert D, Spector SA, Rouphael N, Creech CB, McGettigan J, Khetan S, Segall N, Solis J, Brosz A, Fierro C, Schwartz H, Neuzil K, Corey L, Gilbert P, Janes H, Follmann D, Marovich M, Mascola J, Polakowski L, Ledgerwood J, Graham BS, Bennett H, Pajon R, Knightly C, Leav B, Deng W, Zhou H, Han S, Ivarsson M, Miller J, and Zaks T. (2021). Efficacy and safety of the mRNA-1273 SARS-CoV-2 vaccine. N Engl J Med 384:403–416 33378609 10.1056/NEJMoa2035389 PMC7787219

[B4] Bartoszko JJ, Farooqi MAM, Alhazzani W, and Loeb M. (2020). Medical masks vs N95 respirators for preventing COVID-19 in healthcare workers: a systematic review and meta-analysis of randomized trials. Influenza Other Respir Viruses 14:365–373 32246890 10.1111/irv.12745 PMC7298295

[B5] BBC. (2020). Lake District mountain rescue call-outs rise by 70%. BBC News

[B6] BEAL. (2020). How to disinfect BEAL textile PPE—BEAL. Available at https://www.beal-planet.com/en/how-to-disinfect-beal-textile-ppe (accessed 1227, 2020)

[B7] Berlin DA, Gulick RM, and Martinez FJ. (2020). Severe Covid-19. N Engl J Med 383:2451–2460 32412710 10.1056/NEJMcp2009575

[B8] Binks AC, Parkinson SM, and Sabbouh V. (2020). Oxygen: under or over a surgical facemask for COVID-19 patients? Anaesthesia 75:1691–1692 32525226 10.1111/anae.15166 PMC7307031

[B9] Black Diamond Equipment. (2020). Equipment disinfection recommendations. Black Diamond. Available at https://www.blackdiamondequipment.com/on/demandware.static/-/Library-Sites-SharedLibrary/default/dwb60beab4/tech-pdfs/S20_EquipmentCleaning_17x11_ENG.pdf (accessed 815, 2020)

[B10] Bredmose PP, Diczbalis M, Butterfield E, Habig K, Pearce A, Osbakk SA, Voipio V, Rudolph M, Maddock A, and O'Neill J. (2020). Decision support tool and suggestions for the development of guidelines for the helicopter transport of patients with COVID-19. Scand J Trauma Resusc Emerg Med 28:43 32450877 10.1186/s13049-020-00736-7 PMC7247287

[B11] British Columbia Search and Rescue Association (BCSARA). (2020). Highest number of searches and rescues recorded for July | BC Search and Rescue Association. Available at https://www.bcsara.com/2020/08/highest-number-of-searches-and-rescues-recorded-for-july (accessed 113, 2021)

[B12] Burton C, Coles B, Adisesh A, Smith S, Toomey E, Chan XH, Ross L, and Greenhalgh T. (2020). Performance and impact of disposable and reusable respirators for healthcare workers during pandemic respiratory disease: a rapid evidence review. MedRxiv 2020.05.21.20108233 10.1136/oemed-2020-107058 33504624

[B13] Centers for Disease Control and Prevention (CDC). (2020a). Interim operational considerations for public health management of healthcare workers exposed to or with suspected or confirmed COVID-19: non-U.S. healthcare settings. Centers for Disease Control and Prevention. Available at https://www.cdc.gov/coronavirus/2019-ncov/hcp/non-us-settings/public-health-management-hcw-exposed.html (accessed 115, 2021)

[B14] Centers for Disease Control and Prevention (CDC). (2020b). Interim U.S. guidance for risk assessment and work restrictions for healthcare personnel with potential exposure to COVID-19. Centers for Disease Control and Prevention. Available at https://www.cdc.gov/coronavirus/2019-ncov/hcp/guidance-risk-assesment-hcp.html (accessed 115, 2021)

[B15] Centers for Disease Control and Prevention (CDC). (2020c). Considerations for wearing masks. Centers for Disease Control and Prevention. Available at https://www.cdc.gov/coronavirus/2019-ncov/prevent-getting-sick/cloth-face-cover-guidance.html (accessed 1227, 2020)

[B16] Centers for Disease Control and Prevention (CDC). (2020d). Cleaning and disinfection for non-emergency transport vehicles. Centers for Disease Control and Prevention. Available at https://www.cdc.gov/coronavirus/2019-ncov/community/organizations/disinfecting-transport-vehicles.html (accessed 111, 2021)

[B17] Centers for Disease Control and Prevention (CDC). (2020e). Cleaning and disinfecting your facility: everyday steps, steps when someone is sick, and considerations for employers. Centers for Disease Control and Prevention. Available at https://www.cdc.gov/coronavirus/2019-ncov/community/disinfecting-building-facility.html (accessed 111, 2021)

[B18] Chen Y, Yang Z, Wang J, and Gong H. (2016). Physiological and subjective responses to breathing resistance of N95 filtering facepiece respirators in still-sitting and walking. Int J Ind Ergon 53:93–101

[B19] Chin AWH, Chu JTS, Perera MRA, Hui KPY, Yen H-L, Chan MCW, Peiris M, and Poon LLM. (2020). Stability of SARS-CoV-2 in different environmental conditions. Lancet Microbe 1:e10 32835322 10.1016/S2666-5247(20)30003-3 PMC7214863

[B20] Chou R, Dana T, Jungbauer R, Weeks C, and McDonagh MS. (2020). Masks for prevention of respiratory virus infections, including SARS-CoV-2, in health care and community settings. Ann Intern Med 173:542–555 32579379 10.7326/M20-3213 PMC7322812

[B21] Chu DK, Akl EA, Duda S, Solo K, Yaacoub S, Schünemann HJ, Chu DK, Akl EA, El-harakeh A, Bognanni A, Lotfi T, Loeb M, Hajizadeh A, Bak A, Izcovich A, Cuello-Garcia CA, Chen C, Harris DJ, Borowiack E, Chamseddine F, Schünemann F, Morgano GP, Muti Schünemann GEU, Chen G, Zhao H, Neumann I, Chan J, Khabsa J, Hneiny L, Harrison L, Smith M, Rizk N, Giorgi Rossi P, AbiHanna P, El-khoury R, Stalteri R, Baldeh T, Piggott T, Zhang Y, Saad Z, Khamis A, Reinap M, Duda S, Solo K, Yaacoub S, and Schünemann HJ. (2020). Physical distancing, face masks, and eye protection to prevent person-to-person transmission of SARS-CoV-2 and COVID-19: a systematic review and meta-analysis. Lancet 395:1973–1987 32497510 10.1016/S0140-6736(20)31142-9 PMC7263814

[B22] DMM Wales. (2020). COVID-19: care, cleaning & disinfection of DMM equipment. https://blog-cdn.papertrail.io/wp-content/uploads/2020/04/30144224/DMM-COVID-19-Gear.pdf (accessed 1227, 2020)

[B23] van Doremalen N, Bushmaker T, Morris DH, Holbrook MG, Gamble A, Williamson BN, Tamin A, Harcourt JL, Thornburg NJ, Gerber SI, Lloyd-Smith JO, de Wit E, and Munster VJ. (2020). Aerosol and surface stability of SARS-CoV-2 as compared with SARS-CoV-1. N Engl J Med 382:1564–1567 32182409 10.1056/NEJMc2004973 PMC7121658

[B24] Drozd A, Smereka J, Filipiak KJ, Jaguszewski M, Ładny JR, Bielski K, Nadolny K, Ruetzler K, and Szarpak Ł. (2021). Intraosseous versus intravenous access while wearing personal protective equipment: a meta-analysis in the era of COVID-19. Kardiol Pol 79:277–286 33415967 10.33963/KP.15741

[B25] Ellerton J, Tomazin I, Brugger H, and Paal P. (2009). Immobilization and splinting in mountain rescue. High Alt Med Biol 10:337–342 20039814 10.1089/ham.2009.1038

[B26] Epstein D, Korytny A, Isenberg Y, Marcusohn E, Zukermann R, Bishop B, Minha S, Raz A, and Miller A. (2021). Return to training in the COVID-19 era: the physiological effects of face masks during exercise. Scand J Med Sci Sports 31:70–75 32969531 10.1111/sms.13832 PMC7646657

[B27] Fikenzer S, Uhe T, Lavall D, Rudolph U, Falz R, Busse M, Hepp P, and Laufs U. (2020). Effects of surgical and FFP2/N95 face masks on cardiopulmonary exercise capacity. Clin Res Cardiol 109:1522–1530 32632523 10.1007/s00392-020-01704-y PMC7338098

[B28] Gandhi RT, Lynch JB, and del Rio C. (2020). Mild or moderate Covid-19. N Engl J Med 383:1757–1766 32329974 10.1056/NEJMcp2009249

[B29] Gawel D. (2020). Colorado search and rescue teams see increased volume of missions in 2020. SnowBrains. Available at https://snowbrains.com/colorado-search-and-rescue-teams-have-had-an-increased-volume-of-missions-in-20202 (accessed 113, 2021)

[B30] Greatbatch I, Allen I, and Williams DG. (2020). Decontamination of technical rope rescue equipment in the COVID-19 novel coronavirus pandemic. J Search Rescue 4:200

[B31] Guyatt G, Gutterman D, Baumann MH, Addrizzo-Harris D, Hylek EM, Phillips B, Raskob G, Lewis SZ, and Schünemann H. (2006). Grading strength of recommendations and quality of evidence in clinical guidelines: report from an American College of Chest Physicians Task Force. Chest 129:174–181 16424429 10.1378/chest.129.1.174

[B32] Hopkins SR, Dominelli PB, Davis CK, Guenette JA, Luks AM, Molgat-Seon Y, Sá RC, Sheel AW, Swenson ER, and Stickland MK. (2020). Face masks and the cardiorespiratory response to physical activity in health and disease. Ann Am Thorac Soc 18:399–407 10.1513/AnnalsATS.202008-990CME PMC7919154 33196294

[B33] Hu JC. (2020). Wilderness rescuers brace for a rough Covid-19 winter. WIRED

[B34] Jones JG. (1991). The physiological cost of wearing a disposable respirator. Am Ind Hyg Assoc J 52:219–225 1858664 10.1080/15298669191364631

[B35] Kates J, Moss K, and Oum S. (2020). Preparing for COVID-19 in low- and middle-income countries: leveraging U.S. global health assets. Kaiser Family Foundation. Available at https://www.kff.org/coronavirus-covid-19/issue-brief/preparing-for-covid-19-in-low-and-middle-income-countries-leveraging-u-s-global-health-assets (accessed 1227, 2020)

[B36] Kienbacher CL, Grafeneder J, Tscherny K, Krammel M, Fuhrmann V, Niederer M, Neudorfsky S, Herbich K, Schreiber W, Herkner H, and Roth D. (2021). The use of personal protection equipment does not impair the quality of cardiopulmonary resuscitation: a prospective triple-cross over randomised controlled non-inferiority trial. Resuscitation 160:79–83 33524489 10.1016/j.resuscitation.2021.01.021

[B37] Knibbs LD, Morawska L, and Bell SC. (2012). The risk of airborne influenza transmission in passenger cars. Epidemiol Infect 140:474–478 21733264 10.1017/S0950268811000835

[B38] Kong Italy. (2020). Disinfecting KONG products from SARS-CoV-2. https://www.kong.it/en/15-download/items/media/11-documents/KONG_SARS-CoV-2_DISINFECTION_IT_EN1.pdf (accessed 1227, 2020)

[B39] Krystal JH. (2020). Responding to the hidden pandemic for healthcare workers: stress. Nat Med 26:639 32350461 10.1038/s41591-020-0878-4

[B40] Lan J, Song Z, Miao X, Li H, Li Y, Dong L, Yang J, An X, Zhang Y, Yang L, Zhou N, Yang L, Li J, Cao J, Wang J, and Tao J. (2020). Skin damage among health care workers managing coronavirus disease-2019. J Am Acad Dermatol 82:1215–1216 32171808 10.1016/j.jaad.2020.03.014 PMC7194538

[B41] Leung NHL, Chu DKW, Shiu EYC, Chan K-H, McDevitt JJ, Hau BJP, Yen H-L, Li Y, Ip DKM, Peiris JSM, Seto W-H, Leung GM, Milton DK, and Cowling BJ. (2020). Respiratory virus shedding in exhaled breath and efficacy of face masks. Nat Med 26:676–680 32371934 10.1038/s41591-020-0843-2 PMC8238571

[B42] Liang M, Gao L, Cheng C, Zhou Q, Uy JP, Heiner K, and Sun C. (2020). Efficacy of face mask in preventing respiratory virus transmission: a systematic review and meta-analysis. Travel Med Infect Dis 36:101751 32473312 10.1016/j.tmaid.2020.101751 PMC7253999

[B43] Lindsley WG, Blachere FM, Law BF, Beezhold DH, and Noti JD. (2020). Efficacy of face masks, neck gaiters and face shields for reducing the expulsion of simulated cough-generated aerosols. Aerosol Sci Technol 55:1–9 10.1080/02786826.2020.1862409 PMC9345365 35924077

[B44] Long Y, Hu T, Liu L, Chen R, Guo Q, Yang L, Cheng Y, Huang J, and Du L. (2020). Effectiveness of N95 respirators versus surgical masks against influenza: a systematic review and meta-analysis. J Evid Based Med 13:93–101 32167245 10.1111/jebm.12381 PMC7228345

[B45] MacIntyre CR, Wang Q, Seale H, Yang P, Shi W, Gao Z, Rahman B, Zhang Y, Wang X, Newall AT, Heywood A, and Dwyer DE. (2013). A randomized clinical trial of three options for N95 respirators and medical masks in health workers. Am J Respir Crit Care Med 187:960–966 23413265 10.1164/rccm.201207-1164OC

[B46] Mammut. (2020). Disinfecting of personal protective equipment. Available at https://www.mammut.com/us/en/service/product-service/disinfecting-of-ppe (accessed 1227, 2020)

[B47] Massullo D, Fiorelli S, Rubcich P, Romano D, and Facchetti G. (2021). Mountain rescue during the COVID-19 outbreak: considerations and practical implications. Wilderness Environ Med 32:123–125 33317931 10.1016/j.wem.2020.09.003 PMC7510558

[B48] Maxim Ropes. (2020). Rope disinfection: Covid-19. https://www.maximropes.com/fileadmin/user_upload/Tech_Info/08_Rope_disinfection/20-03-23_Corona_Rope_Disinfection_MAXIM_EN.pdf (accessed 1227, 2020)

[B49] Miguel-Puga JA, Cooper-Bribiesca D, Avelar-Garnica FJ, Sanchez-Hurtado LA, Colin-Martínez T, Espinosa-Poblano E, Anda-Garay JC, González-Díaz JI, Segura-Santos OB, Vital-Arriaga LC, and Jáuregui-Renaud K. (2021). Burnout, depersonalization, and anxiety contribute to post-traumatic stress in frontline health workers at COVID-19 patient care, a follow-up study. Brain Behav 11:e02007 33319496 10.1002/brb3.2007 PMC7883101

[B50] Mushtaq S, Terzi E, Recalcati S, Salas-Alanis JC, Amin S, and Faizi N. (2021). Cutaneous adverse effects due to personal protective measures during COVID-19 pandemic: a study of 101 patients. Int J Dermatol 60:327–331 33320331 10.1111/ijd.15354

[B51] Nolan JP, Monsieurs KG, Bossaert L, Böttiger BW, Greif R, Lott C, Madar J, Olasveengen TM, Roehr CC, Semeraro F, Soar J, Van de Voorde P, Zideman DA, Perkins GD, Ainsworth S, Biarent D, Bingham B, Blom MT, Borra V, Bossaert L, Böttiger BW, Brissaud O, Carli P, Cassan P, Castrén M, Cimpoesu D, Couper K, Deakin CD, Buck ED, Lucas ND, Djakow J, Djärv T, Druwé P, Ersdal H, Handley A, Hoffmann F, Klaassen B, Kuzovlev A, Lauritsen T, Lilja G, Lott C, Lulic I, Maconochie I, Madar J, Martinez AM, Mentzelopoulos S, Meyran D, Monsieurs KG, Morley C, Nolan JP, Olasveengen T, Paal P, Pellis T, Perkins GD, Raffay V, Ristagno G, Roehr C, Rüdiger M, Sandroni C, Semeraro F, Singletary E, Skåre C, Smyth M, Soar J, Svavarsdóttir H, Szczapa T, Pas A te, Trevisanuto D, Turner NM, Urlesberger B, Voorde PV de, Wilkinson D, Wyllie J, and Zideman DA. (2020). European Resuscitation Council COVID-19 guidelines executive summary. Resuscitation 153:45–55 32525022 10.1016/j.resuscitation.2020.06.001 PMC7276132

[B52] Osborn L, Meyer D, Dahm P, Ferguson B, Cabrera R, Sanger D, Mock M, Herrera T, Mader S, and Ostrosky-Zeichner L. (2020). Integration of aeromedicine in the response to the COVID-19 pandemic. J Am Coll Emerg Physicians Open 1:557–562 10.1002/emp2.12117 PMC7283804 32838374

[B53] Paal P, Gordon L, Strapazzon G, Brodmann Maeder M, Putzer G, Walpoth B, Wanscher M, Brown D, Holzer M, Broessner G, and Brugger H. (2016). Accidental hypothermia–an update. Scand J Trauma Resusc Emerg Med 24:111 27633781 10.1186/s13049-016-0303-7 PMC5025630

[B54] Perkins GD, Morley PT, Nolan JP, Soar J, Berg K, Olasveengen T, Wyckoff M, Greif R, Singletary N, Castren M, de Caen A, Wang T, Escalante R, Merchant RM, Hazinski M, Kloeck D, Heriot G, Couper K, and Neumar R. (2020). International Liaison Committee on Resuscitation: COVID-19 consensus on science, treatment recommendations and task force insights. Resuscitation 151:145–147 32371027 10.1016/j.resuscitation.2020.04.035 PMC7194051

[B55] Petzl America. (2020). Recommendations for disinfecting your equipment. Available at https://www.petzl.com/INT/en/Professional/News/2020-4-17/Recommendations-for-disinfecting-your-equipment (accessed 1227, 2020)

[B56] Pradhan D, Biswasroy P, Kumar Naik P, Ghosh G, and Rath G. (2020). A review of current interventions for COVID-19 prevention. Arch Med Res 51:363–374 32409144 10.1016/j.arcmed.2020.04.020 PMC7190516

[B57] Qian H, Miao T, Liu L, Zheng X, Luo D, and Li Y. (2021). Indoor transmission of SARS-CoV-2. Indoor Air 31:639–645 33131151 10.1111/ina.12766

[B58] Radonovich LJ Jr., Simberkoff MS, Bessesen MT, Brown AC, Cummings DAT, Gaydos CA, Los JG, Krosche AE, Gibert CL, Gorse GJ, Nyquist A-C, Reich NG, Rodriguez-Barradas MC, Price CS, and Perl TM; for the ResPECT Investigators. (2019). N95 respirators vs medical masks for preventing influenza among health care personnel: a randomized clinical trial. JAMA 322:824–833 31479137 10.1001/jama.2019.11645 PMC6724169

[B59] Sahin U, Muik A, Derhovanessian E, Vogler I, Kranz LM, Vormehr M, Baum A, Pascal K, Quandt J, Maurus D, Brachtendorf S, Lörks V, Sikorski J, Hilker R, Becker D, Eller A-K, Grützner J, Boesler C, Rosenbaum C, Kühnle M-C, Luxemburger U, Kemmer-Brück A, Langer D, Bexon M, Bolte S, Karikó K, Palanche T, Fischer B, Schultz A, Shi P-Y, Fontes-Garfias C, Perez JL, Swanson KA, Loschko J, Scully IL, Cutler M, Kalina W, Kyratsous CA, Cooper D, Dormitzer PR, Jansen KU, and Türeci Ö. (2020). COVID-19 vaccine BNT162b1 elicits human antibody and TH1 T cell responses. Nature 586:594–599 32998157 10.1038/s41586-020-2814-7

[B60] Scottish Mountain Rescue. (2020). Mountain Rescue and COVID—how have we adapted and are finding solutions for the longer pandemic…. Scottish Mountain Rescue. Available at https://www.scottishmountainrescue.org/mountain-rescue-and-covid-how-have-we-adapted-and-are-finding-solutions-for-the-longer-pandemic (accessed 113, 2021)

[B61] Shaw J, Day T, Malik N, Barber N, Wickenheiser H, Fisman DN, Bogoch I, Brownstein JI, and Williamson T. (2020). Working in a bubble: how can businesses reopen while limiting the risk of COVID-19 outbreaks? CMAJ 192:E1362–E1366 32998942 10.1503/cmaj.201582 PMC7647488

[B62] Singing Rock. (2020). COVID-19 disinfection statement. https://blog-cdn.papertrail.io/wp-content/uploads/2020/04/17095236/SINGING_ROCK_OFFICIAL_DISINFECTION_PPE_STATEMENT.pdf (accessed 1227, 2020)

[B63] Strapazzon G, Reisten O, Argenone F, Zafren K, Zen-Ruffinen G, Larsen GL, and Soteras I. (2018). International Commission for Mountain Emergency Medicine Consensus guidelines for on-site management and transport of patients in canyoning incidents. Wilderness Environ Med 29:252–265 29422373 10.1016/j.wem.2017.12.002

[B64] Tricco AC, Lillie E, Zarin W, O'Brien KK, Colquhoun H, Levac D, Moher D, Peters MDJ, Horsley T, Weeks L, Hempel S, Akl EA, Chang C, McGowan J, Stewart L, Hartling L, Aldcroft A, Wilson MG, Garritty C, Lewin S, Godfrey CM, Macdonald MT, Langlois EV, Soares-Weiser K, Moriarty J, Clifford T, Tunçalp Ö, and Straus SE. (2018). PRISMA Extension for scoping reviews (PRISMA-ScR): checklist and explanation. Ann Intern Med 169:467–473 30178033 10.7326/M18-0850

[B65] Wang X, Pan Z, and Cheng Z. (2020). Association between 2019-nCoV transmission and N95 respirator use. J Hosp Infect 105:104–105 32142885 10.1016/j.jhin.2020.02.021 PMC7134426

[B66] Wiersinga WJ, Rhodes A, Cheng AC, Peacock SJ, and Prescott HC. (2020). Pathophysiology, transmission, diagnosis, and treatment of coronavirus disease 2019 (COVID-19): a review. JAMA 324:782–793 32648899 10.1001/jama.2020.12839

[B67] World Health Organization, Department of Food Safety, Zoonoses and Foodborne Diseases. (2006). Five keys to safer food manual. WHO Press, France

[B68] World Health Organization (WHO). (2020a). Risk assessment and management of exposure of health care workers in the context of COVID-19: interim guidance, 19 March 2020. World Health Organization, Geneva

[B69] World Health Organization (WHO). (2020b). Mask use in the context of COVID-19—interim guidance. Emergencies Preparedness, WHO Headquarters. World Health Organization, Geneva

[B70] World Health Organization (WHO). (2020c). Rational use of personal protective equipment for COVID-19 and considerations during severe shortages: interim guidance, 23 December 2020. World Health Organization, Geneva

[B71] World Health Organization (WHO). (2020d). Coronavirus disease (COVID-19): Health and safety in the workplace. World Health Organization. Available at https://www.who.int/news-room/q-a-detail/coronavirus-disease-covid-19-health-and-safety-in-the-workplace (accessed 1226, 2020)

[B72] World Health Organization (WHO). (2020e). COVID-19 and food safety: guidance for food businesses. World Health Organization, Geneva

[B73] World Health Organization (WHO). (2021). COVID-19 strategic preparedness and response plan (SPRP 2021). World Health Organization—Publications. Available at https://www.who.int/publications/i/item/covid-19-strategic-preparedness-and-response-plan-(sprp-2021) (accessed 227, 2021)

[B74] Yaacoub S, Schünemann HJ, Khabsa J, El-Harakeh A, Khamis AM, Chamseddine F, El Khoury R, Saad Z, Hneiny L, Cuello Garcia C, Muti-Schünemann GEU, Bognanni A, Chen C, Chen G, Zhang Y, Zhao H, Abi Hanna P, Loeb M, Piggott T, Reinap M, Rizk N, Stalteri R, Duda S, Solo K, Chu DK, and Akl EA. (2020). Safe management of bodies of deceased persons with suspected or confirmed COVID-19: a rapid systematic review. BMJ Glob Health 5:e002650 10.1136/bmjgh-2020-002650 PMC7234869 32409328

[B75] Zafren K, McCurley LH, Shimanski C, and Smith W. (2012). Technical rescue, self rescue, and evacuation. In: Wilderness Medicine. PS Auerbach, ed. Elsevier, Philadelphia, PA. 457–463

