# Supplementary material for: Guidelines for Mountain Rescue During the COVID-19 Pandemic: Official Guidelines of the International Commission for Alpine Rescue
Source: High Alt Med Biol. 2021 Jun 23;22(2):128–41. doi: 10.1089/ham.2021.0032 (PMC8252899; doi:10.1089/ham.2021.0032)
Supplement: Supplemental data [file Supp_AppS1.pdf]

# ICAR Guidelines for Mountain Rescue during the COVID-19 Pandemic

Official Guidelines of ICAR MEDCOM

Appendix 1 - Rescuer Summary

The **ICAR Guidelines for Mountain Rescue during the COVID-19 Pandemic** covers several important topics pertaining to mountain rescue.

This document is an abridged, plain language summary of key ‘take-aways’ from the ICAR MedCom guidelines. The full MedCom guidelines are being published in High Altitude Medicine and Biology, and should be referred to for more detail and explanation.

Statements by professional rescuers are included throughout the manuscript quoted in *italics* to highlight how the COVID-19 pandemic is challenging rescue teams. The recommendations are numbered in keeping with the full text guidelines and are followed by a strength grading which is based on the evidence grading system of the American College of Chest Physicians (see full text for details).

## Rescuer safety and infection prevention

A significant issue during the pandemic has been the speed that the virus has spread and the evidence-base on how to deal with it. The accompanying national and regional regulation changes have been equally rapid and rarely had chance to consider the special circumstances of mountain rescue. Hence the following sentiment from a professional rescuer:

> “*We need to be careful that we are not just moving risk around if the true goal is to reduce the risk. Wearing masks, gloves and full personal protective equipment (PPE) in technical terrain creates its own hazards. It may reduce the potential for a COVID-19 exposure but the potential for a fall or mistake in technical terrain is increased.*”

The risk of contracting COVID-19 outdoors is low when rescuers are careful and follow hand washing and social distancing measures. Increasing levels of protection should be instigated before you are in close contact with patients and other rescuers. During technical rescue activities, PPE may introduce additional risk as a result of unacceptable communication or visibility. Limits on exertion and performance can impair a rescue. In this way, COVID-19 precautions may complicate, distract from or increase some risks of mountain rescue.

> *As with many things, we start the day by following a procedure and as people get tired or it becomes a bit cumbersome our precautions tend to drop off.*

**Recommendation 1, 2 & 5**

Mountain Rescuers must maintain situational awareness by identifying and managing the risks of exposure to COVID-19 during mountain rescue in the context of the general risks of mountain rescue. (1C)

**Recommendation 3, 4 & 66**

Any mountain rescuer that is ill with symptoms of COVID-19, or has been advised to self-isolate following a positive COVID-19 test or through contact tracing, must not respond to missions and must not participate in training exercises. (1C)

**Recommendation 6**

Mountain rescuers should be trained in the selection, putting on (‘donning’) and removing (‘doffing’) of appropriate PPE. Refreshing, updating and alternative techniques should be considered to encourage continued compliance with the recommendations. (2C)

**Recommendation 1 & 7**

Mountain rescuers should follow appropriate national, regional and organisational instruction on the wearing of face coverings. Outdoors, a medical grade surgical mask covering mouth and nose should be worn whenever there is close contact (≤ 2 meters) with other rescuers or patients, with the proviso that it does not introduce other unacceptable safety risks. (1A)

**Recommendation 23 & 25**

A surgical mask should be placed on the patient immediately upon contact and kept in place throughout the rescue and evacuation. An oxygen mask can be placed on top of this. (2C)

**Recommendations 8, 13, 14, & 24**

Mountain rescuers that may be involved in close contact with patients during first aid and medical procedures should don PPE before encroaching within 2 meters of the patient. They should wear masks with N95/FFP2 or higher ratings. (1A) They must wear eye protection and waterproof medical grade gloves. (1C)

**Recommendations 11 & 12**

Water resistant masks should be considered in some environments and wet masks should be changed as soon as possible. (2C)

**Recommendation 15**

Hand washing must be performed before donning and after doffing PPE; before and after patient care, before and after eating or touching one's face, after blowing your nose, coughing, or sneezing, and after cleaning or disinfecting equipment. If soap and water are not readily available, use a hand sanitizer that contains at least 70% alcohol. (1C)

> *"Personally, I know I haven't been cleaning my own equipment as much as I should be."*

**Recommendation 16 & 19**

Potentially contaminated rescue and personal equipment must be cleaned with soap and water, 70% isopropyl alcohol, or viricidal agent. The manufacturer's instructions should be followed to avoid damage to the material. (1C) If cleaning is not possible, the equipment can be quarantined for a minimum of 72 hrs or disposed in an appropriate way. (2C)

**Recommendation 20 & 21**

COVID-19 vaccination is recommended for eligible mountain rescuers. (1A) Vaccination may not not remove the need for the individual safety precautions recommended above. (2C)

**Recommendation 22**

Mountain rescue teams should encourage and promote communication and training activities that address rescuer stress and promote psychological resilience. (2C)

> *"Because I spend so much time with my team and I have such a high level of trust, I find my COVID-19 precautions slipping at times during training."*

**Recommendation 62, 64 & 65**

Mountain Rescue Organisations may need to adjust their first aid and medical advice in order to minimising the chance of COVID-19 transmission. Any changes must be clearly communicated in a timely fashion to rescuers. Support and training should be given, especially when long-held practices (for example, in CPR) are modified. (2C)
